# Land gradient and configuration effects on yield, irrigation amount and irrigation water productivity in rice-wheat and maize-wheat cropping systems in Eastern India

**DOI:** 10.1016/j.agwat.2021.107036

**Published:** 2021-09-01

**Authors:** Krishna Prasad Devkota, Sudhir Yadav, E. Humphreys, Akhilesh Kumar, Pankaj Kumar, Virender Kumar, R.K. Malik, Amit K. Srivastava

**Affiliations:** a International Rice Research Institute (IRRI), Los Baños, Laguna, The Philippines; b African Sustainable Agriculture Research Institute (ASARI), Mohammed VI Polytechnic University (UM6P), Laayoune, Morocco; c Griffith, NSW 2680, Australia; d International Maize and Wheat Improvement Center (CIMMYT), Patna, India

**Keywords:** Raised beds, Slope, Advance time, Intake opportunity time, Mechanical transplanting, Bihar

## Abstract

Laser land levelling is expanding rapidly in the rice-wheat (RW) and maize-wheat (MW) systems of the Indo-Gangetic Plains of India and Pakistan. Current practice is to level to zero (0%) gradient, whereas a small gradient (e.g. 0.1%) is typically used in developed countries. Therefore, experiments were conducted in farmers’ plots (~15 m x 40 m) in the Eastern Gangetic Plains to evaluate laser levelling with a 0.1% gradient in comparison with 0% and farmer levelling practice (FL). The study was conducted over two years in RW and MW systems. In the MW system, raised beds in plots lasered with 0% and 0.1% gradients were also evaluated. Laser levelling with 0% gradient significantly reduced irrigation amount and/or increased irrigation water productivity (WPi) in all crops/systems grown on the flat compared to FL except for wheat in the MW system. While there was a consistent trend for higher yield with a 0% gradient compared with FL, the differences were not significant in any crop/system. For the RW system, the results suggest no to marginal benefits in irrigation amount and WPi from levelling with a 0.1% gradient in comparison with 0% gradient. In that system, by far the bigger gains were from changing from FL to laser levelling with 0% gradient. This resulted in substantial reductions in irrigation amount, which greatly increased WPi in both crops (by ~40%), while yield was not affected. Rice grown with FL was not profitable, but lasering with 0% gradient significantly increased gross margin for rice, wheat and the total RW system. As for the RW system, levelling to 0% with a flat configuration significantly increased WPi of both crops in the MW system compared to FL, but by a lesser proportion. Raised beds significantly increased yield of maize by 8% (0.5 t ha^−1^), reduced irrigation amount by 20% (40 mm) and increased WPi by 34% (1.0 kg m^−3^) in comparison with the laser levelled flat plots. Gross margin of the MW system on beds was 17–20% higher than FL, and gross margin with beds on a 0.1% gradient was significantly higher than either gradient on the flat. The results suggest that the gains from levelling with a 0.1% gradient compared to 0% are marginal; however, this may change if the goal of consolidation of small farmer plots into larger fields becomes a reality provided there is a proportionate increase in irrigation flow rates, and ability to drain.

## Highlights

• Land gradient (0, 0.1%) and configuration (flats, beds) were tested in non-puddled rice-wheat (RW) and maize-wheat (MW) systems.
• 0% gradient with flat configuration did not increase yield but increased gross margin of RW system than farmer practice.
• 0.1% gradient with flats increased yield, WPi and gross margin of all crops compared with farmer levelling.
• In MW, beds increased WPi of maize over flats with the same gradient, and had the highest gross margin regardless of gradient.
• Gross margin of the MW system was 2–4 times that of the RW system.

## 1 Introduction

Sustainability of the annual growth in rice and wheat production is one of the prime concerns for India to feed its fast-growing population. Given the limited prospects for substantial and sustainable increases in crop production in northwest India, the home of the green revolution, the country has policies and/or programs for a second green revolution in eastern India (Pathak et al., 2019). Key attributes of eastern India are large yield gaps and large and untapped groundwater resources, suggesting high potential to greatly increase crop productivity to help feed the nation (Mohanty et al., 2017).

Laser-assisted land levelling (“laser levelling”) is one technology helping to reduce the yield gap, and its use is expanding rapidly in the Indo-Gangetic Plains (IGP) of South Asia, with 1.5 Mha laser levelled by 2015 (Jat et al., 2015). In the IGP, irrigated rice-wheat (RW) systems predominate, while maize-wheat (MW) systems are becoming increasingly important, especially in the eastern IGP. The reasons for the popularity of laser levelling include: (i) reduced irrigation amount (Mathankar et al., 2005, Abdullaev et al., 2007), and improved uniformity of water application and consequently yield (Aryal et al., 2015, Jat et al., 2015), (ii) increased cultivable land area through the reduction of bunds and channels (Naresh et al., 2014), (iii) reduced weed density (in rice) (Rickman, 2002), (iv) increased input-use (fertilizer, pesticide etc.) efficiency (Jat et al., 2011), and (v) more efficient machinery operations and improved crop establishment (Jat et al., 2006). In the RW systems of Pakistan, adoption of laser land levelling has been shown to have positive impacts on wheat and rice yields and household income (Ali et al., 2018).

In the IGP, the fields are laser levelled to create a zero gradient both across and ‘down’ (from head to tail) the field. In contrast, in developed countries, a small gradient (e.g. 0.08–0.2% in 100–700 m long fields in parts of Australia) is common for irrigated upland crops grown in both flat (“border-check”) and bed layouts (Carroll et al., 1995, Khanna et al., 2003, Hamilton et al., 2005). For rice-upland systems in Australia “lasered-contour” layouts with a small gradient (0.4–0.5%) across the plots (perpendicular to the contours) are often used to facilitate drainage. A gradient from head to tail produces more rapid advance of the waterfront down the field, reducing the duration of each irrigation, drainage beyond the bottom of the root zone (“deep drainage”), and waterlogging. An optimal gradient can reduce irrigation amount in upland crops by up to 20% Walker et al., 2006, Bautista et al., 2009, González et al., 2011). Irrigation duration and drainage losses are also strongly influenced by inlet flow rate, cut-off time, length and area of the field, and soil type (Smith and Uddin, 2020). All these factors need to be considered in designing layouts and irrigation management that provide sufficient irrigation water intake opportunity time to wet the root zone across the field as uniformly as possible while minimizing deep drainage losses (Playan et al., 1996, Playán and Mateos, 2006, Walker et al., 2006). While the benefits of laser levelling with zero gradient in both directions have been well-established in the small irrigation plots used by farmers in the IGP (Jat et al., 2015), whether levelling with a small gradient would confer further benefits in terms of yield and/or irrigation water productivity (WPi) has not been considered. In the study area of Bihar state in eastern India, field size is typically 1 acre (64 m × 64 m). For rice, the field is divided into irrigation plots (separated by earthen bunds) of width typically ranging from 10 to 30 m and length from 20 to 60 m. For wheat, the rice plots are sub-divided into shorter and narrower bunded plots, aiming for more efficient irrigation.

In the eastern Gangetic Plains, rice, wheat and maize are the major food and fodder crops. Rice and maize are grown during the monsoon season (June to September), mostly on rainfall but with supplemental irrigation. Maize is also grown in the relatively dry rabi (winter) season in some pockets of the region. Unreliable amounts and distribution of rainfall adversely affect the timeliness of establishment of rice and thus the subsequent wheat crop (grown in winter), reducing the yield of both crops (Balwinder-Singh et al., 2019). In addition, periods of water deficit stress, especially increased terminal drought stress resulting from delayed transplanting, reduce yield in rice (Balwinder-Singh et al., 2019). In this region, rainfall has decreased during the period of rice establishment (July and August), and rainfall variability has increased, over the past 30 years (Subash et al., 2011, Naidu et al., 2017). Puddling and transplanting of rice are delayed until the start of the monsoon rains due to the late arrival (after the start of the monsoon) of low-cost canal water and the high cost of pumping groundwater. Farmers are faced with the choice of late transplanting with old seedlings and thus reduced yield or leaving the field fallow. Options for more timely establishment of crops and lower irrigation amount for crop establishment include mechanical transplanting of rice into non-puddled soil (NP-MTR) (Yadav et al., 2017; Kar et al., 2018), and switching to maize (Kumar et al., 2018). It is also well-established that the use of safe alternate wetting and drying water management for rice (AWD) reduces irrigation amount while maintaining yield, noting that a short period (e.g. 10–15 days) of ponding immediately after transplanting is recommended (Rejesus et al., 2011, Lampayan et al., 2015). Zero tillage (ZT) and planting of both wheat and maize on raised beds have also been shown to reduce irrigation amount while maintaining or increasing yield in the eastern IGP (Jat et al., 2019, Singh et al., 2020) as in other parts of the IGP (Ladha et al., 2008).

Given the above, a study was conducted to determine whether laser levelling with a small gradient would confer additional benefits in RW and MW systems implemented using the most promising technologies for the region, i.e. ZT for wheat in both systems, NP-MTR grown with AWD in the RW system, and raised beds in the MW system. The hypotheses were: (i) laser levelling with a small (0.1%) gradient will increase the rate of advance of the wetting front and reduce irrigation input to all crops compared with zero gradient, (ii) laser levelling with 0.1% gradient will not affect rice yield, (iii) laser levelling with 0.1% gradient will increase the yield of maize and wheat crops grown in flat configurations (because of reduced waterlogging), (iv) raised beds will increase the yield of maize and wheat and reduce irrigation input, and (v) laser levelling with 0.1% gradient will not affect the yield of crops grown on beds. Thus, the objectives of this study were to determine the effects of land gradient (slope) and configuration (bed and flat) on the intake opportunity time, irrigation amount, yield and irrigation water productivity (WPi) of rice, wheat, and maize crops grown in RW and MW cropping systems in eastern India.

## 2 Materials and methods

### 2.1 Experimental sites and soil

**Fig. 1:**
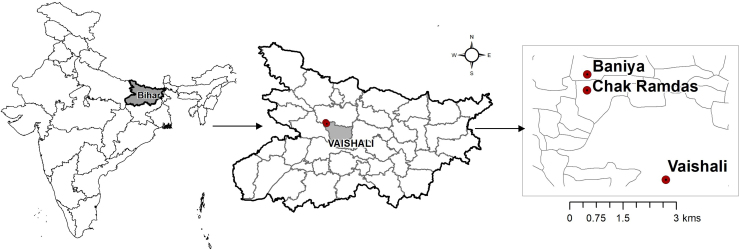
Location of the rice-wheat and maize-wheat systems experiments in Chak Ramdas, Bania and Vaishali villages, Vaishali, Bihar, 2014–2016.

On-farm experiments were conducted from 2014 to 2016 in three neighbouring villages (Vaishali, Chak Ramdas and Bania) in Vaishali district of Bihar state in India (Fig. 1). The villages are located around 26° 00′ N latitude and 85° 06′ E longitude. The experiments were conducted in collaboration with the local non-government organization Vaishali Area Small Farmers’ Association (VASFA), which was engaged in installing deep tubewells and promoting laser land levelling and improved crop establishment methods.

Vaishali district is located within an extensive alluvial plain deposited by the Ganga and the Gandak rivers. Ash-grey-silt/silt-clay/clayey-silt soils predominate and can be broadly grouped into two categories, entisols and inceptisols. The texture of the soil in the experimental plots is clay loam with varying contents of sand and silt (Table S1). Mean volumetric soil water content of the top soil (0–30 cm) at field capacity (10 kPa is 32 cm^3^ cm^−3^). The climate of the region is sub-tropical to sub-humid, with a rainy season from mid-June to the end of September during which about 85% of annual rainfall is received (Tarafdar and Agrawal, 2013). All fields had a long history of growing puddled transplanted rice followed by conventional tillage wheat.

### 2.2 Experimental design and treatments

**Fig. 2:**
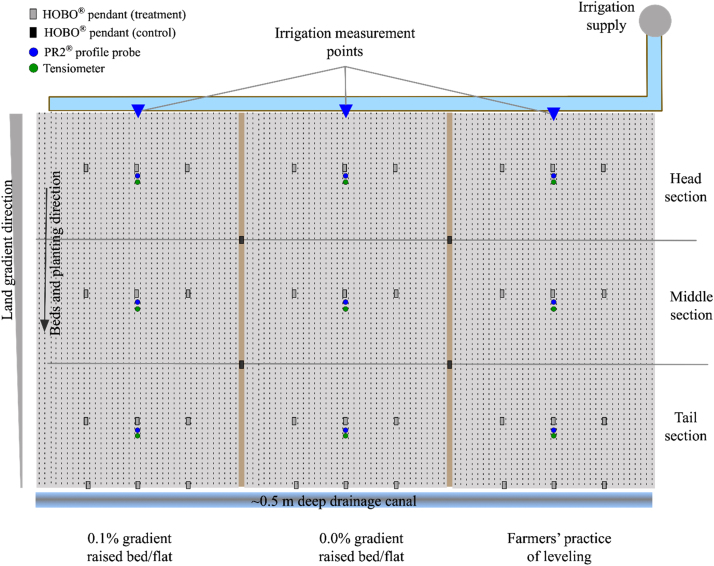
Plot layout and location of instruments (Stingray® flow metres, HOBO® Pendants and grid pegs, PR2® tubes, tensiometers. Figure shows 3 adjacent plots (number of adjacent plots was 3 in RW and 5 in MW).

Two researcher-led on-farm experiments, one each for the RW and MW systems, were conducted for two years (2014–2016), commencing with rice or maize grown during the rainy season in 2014. Each treatment was replicated three times, an individual farmer’s field serving as one replicate. In the RW system, three land gradient treatments (0.0% and 0.1% gradient created by laser levelling, and farmers’ practice of levelling - FL) were compared (Fig. 2). In the MW system, four treatments with combinations of two laser levelled gradients (0% and 0.1%) and two “configurations” (raised beds, flat) were compared with FL. The fields were re-levelled prior to each rainy season crop, to ensure that slope was retained.

### 2.3 Crop management

#### 2.3.1 Land preparation and crop establishment

The fields to be laser levelled were dry ploughed three times using tyne cultivators before creating the land gradients. All implements were drawn by a 55 hp 4-wheel tractor. The gradient was verified using a laser land leveller receiver and transmitter after completion of land levelling. Plot size varied across replicates (i.e. farmers’ fields, 32–46 m long, 14–16 m wide), so the 0.1% gradient resulted in 3.2–4.6 cm difference in elevation between the head (irrigation inlet) and the tail ends of individual plots. Laser levelling was done each year prior to establishment of the rice or maize. For the MW system, raised beds (67 cm between the centres of adjacent beds, width across the flat bed top 37 cm, furrow width 30 cm, furrow depth 15 cm) were formed after laser levelling using a bed shaper. Preparation of fields for FL rice and maize typically involved two dry soil ploughings using a tyne cultivator with a trailing wooden plank, drawn by a 55 hp 4-wheel tractor. There was no puddling in any of the rice treatments and there was no tillage prior to sowing wheat in any treatment in both systems.

Details of field layout, crop establishment and management in the lasered treatments are provided in Fig. 2 and Table S2, and briefly summarized here. Within each crop, the method of establishment was the same across all treatments, both laser levelled and FL, in both the RW and MW systems. Row direction in all plots was from head to tail i.e. in the direction of the slope. For rice, the fields were irrigated, and 15-day old seedlings were planted in the shallow-flooded soil using a six-row transplanter with 20 cm × 20 cm spacing. Wheat was sown at 120 kg ha^−1^ using an 11-row seed-cum-fertilizer-zero-till-drill with row spacing of 18 cm in both flat and bed plots. The T-openers sowing in the slopes of the beds were adjusted so that the seed was also buried. The maize was dibbled manually following a pre-sowing irrigation and a pre-sowing application of glyphosate. Row spacing was 67.5 cm, plant spacing 20 cm, and in the bed plots the maize was planted in the middle of the bed tops. A spade was used to create a slot in the moist soil into which the seed was dropped, and the slot was closed by gently pressing with foot or spade.

#### 2.3.2 Crop management

For rice and maize, hybrid varieties were used, while a long duration inbred variety of wheat was used in both systems (Table S2). The same variety and recommended fertilizer rates and sowing/transplanting, weed and pest management were used in all treatments within each crop. All the phosphorus (P) and potassium (K) fertilizer were applied at the time of transplanting rice or sowing maize and wheat, except that the K fertilizer was broadcast with the 1st topdressing of nitrogen (N) fertilizer in wheat. The P was applied as DAP, and the top-dressed N was applied as urea in 3 splits in rice and two splits in wheat and maize. Weeds were well-controlled through the use of herbicides and manual weeding in rice and maize. In wheat, one application of the herbicide, sulfosulfuron + metsulfuron methyl (Total®), controlled most of weed species, including *Phalaris minor*, broadleaf weeds, and other narrow-leaf weeds and sedges including *Cyperus* species.

The crops were irrigated by pumping groundwater from a supply canal at the head of the plots, through a pipe (diameter 75 mm) in the centre of the bund. The pumping rate was adjusted during each irrigation of individual plots to achieve a constant flow rate during and across irrigations. The discharge rate of the pump was higher during irrigation of rice (12.6±4.0 L s^−1^) than wheat (7.1±1.8 L s^−1^) in the RW system. In the MW system, discharge rates were similar for maize (5.9±1.9 L s^−1^) and wheat (6.6±1.9 L s^−1^). Irrigations were scheduled according to soil water tension. One tensiometer was installed near the centre of the head, mid and tail sections of each plot (Fig. 2), at 15 cm soil depth in rice, and at 32.5 cm in wheat and maize. The tensiometers were read daily. The crops were irrigated within 1–2 days after average soil water tension had increased to 10, 35 and 50 kPa in rice, wheat and maize, respectively (Fig. S1). Irrigation was ceased (“cut-off”) as soon the irrigation front (the water flowing over the soil surface) reached a peg at the mid-point of the bottom edge of the plot. The same criteria for irrigation scheduling and cut-off were applied to all treatments within each crop each year. There was no back up of drainage water in the ditch at the tail end of the experimental plots, as the water was able to drain freely into the end of the adjacent farmers’ field at each site, which was 10–50 cm lower in the landscape.

In typical farmers’ practice, irrigation plot size for rice in this region ranges from 20 to 60 m length and 10–30 m width, and the fields are puddled then levelled with wooden plank drawn by a tractor. For wheat the rice plots are sub divided into 4 sections. The plots are typically flood-irrigated using groundwater delivered to individual plots using flexible pipe. The groundwater is pumped using 6.5 hp diesel operated pumps, giving discharge rates into plots of 4–17 L s^−1^ depending on the distance from the plot and hydraulic head. Generally, farmers re-irrigate rice (depending on water availability and affordability) when the soil surface begins to crack (i.e. the cracks could be larger than “hairline”), and add enough water to achieve a standing water depth of 5–15 cm. Three to five (3−5) irrigations are common for rice. For wheat, 2–4 irrigations are common, applied at crown root initiation, N fertilizer topdressing (twice) and flowering (if four), with plots flooded to a depth of 3–5 cm. For rainy season maize, 1–2 irrigations are generally applied (at seedling and tassling/silking), the need for the later irrigation based on temporary wilting of the upper leaf, with the soil flooded to 3–5 cm depth.

### 2.4 Measurements and observations

#### 2.4.1 Weather data

Daily rainfall and air temperature were extracted from ERA5 daily aggregates using the Google Earth Engine (GEE) (rain gauges were installed in 3 locations, but gaps in the data necessitated an alternative approach). This approach provides data at 0.25 arc degrees, equivalent to 25 km resolution at the latitude of Vaishali. Daily climate reanalysis parameters on total precipitation and mean 2 m air temperature (both at 0.25 arc degree) of ERA5, which is the fifth-generation atmospheric reanalysis of ECMWF (European Centre for Medium-Range Weather Forecasts) global climate, were extracted using the GEE. In GEE, image collection for precipitation and temperature layers from ERA5 was defined, a geographical location of the study area was assigned with date range filter, and data were extracted as csv files. Daily solar radiation data of the same time range were downloaded from the NASA-POWER (National Aeronautics and Space Administration – Prediction of Worldwide Energy Resources) website, which provides data at 1 arc degree spatial resolution.

#### 2.4.2 Grain yield

Grain yield was determined from areas harvested at six locations in each plot, two within each of the head, middle and tail sections. Rice and wheat were harvested from 4 m^2^ quadrats, and maize from 9 m^2^ at each of the 6 locations. For all crops, grain yield was expressed in t ha^−1^ at 14% moisture content (determined by oven drying the grain at 65 °C for 72 h).

#### 2.4.3 Water advance, recession, and intake opportunity time

##### 2.4.3.1 Rice and maize

Twelve bamboo sticks were installed to form a rectangular grid in each plot for monitoring the advance and recession of the irrigation water. The plots were divided into three sections (head, middle, tail) of equal length, and three sticks were placed at positions ¼, ½ and ¾ the way across the middle of each section. An additional row of three sticks was placed near the end of the plots, 1 m from the bund. One HOBO® Pendant Temp/Alarm (‘pendant’) was also placed at each grid point (‘spot’) (Fig. 2). For bed plots, the pendants were placed in the furrows. In addition, two pendants were placed on the bund of each plot to determine air temperature (control temperature). All pendants and the stopwatch (for manual determination) used for determining time when the water reached or receded from each monitoring spot were synchronized for time with the same computer. The HOBO® pendant used in this study measures temperature, and the observation interval was set to one minute (min). During the rice/maize season, the air temperature is high. As the water touches the pendant, its temperature decreases; when the water drains away (recedes) from the pendant, its temperature comes closer to air temperature. The times of advance and recession were thus determined based on the temperature difference between the pendants in the irrigated plot and the pendants on the bund. To reduce the influence of soil temperature on the pendants, a thin and pendant-sized piece of plywood was attached tightly with a rubber band to ensure that the determination of the advance and recession was based solely on the difference in the water and air temperatures. In all plots, inlet time (the time water first entered the plot) was recorded manually. The water advance and recession times computed using the pendant were checked against visual observations using the bamboo grid marks and stopwatch. The average of the three pendants across the plot was used to determine both advance and recession times at each of the four locations along the plot (head, middle, tail, end).

##### 2.4.3.2 Wheat

Visual observations were used to determine advance and recession times during irrigation of the wheat crops, as air temperature was lower than the water temperature in winter.

The water advance rate was computed for all irrigations of all crops in both years. The time of recession was also determined for all irrigations of wheat and maize, and for 7 irrigations of rice each year. Intake opportunity time was computed by subtracting the time for water advance from the time of recession at each of the four locations along each plot. As plot length varied from 32 to 46 m, the advance and recession data were normalized for a plot length of 40 m, with the centres of the head, middle, tail and end at 6.7, 20, 33.3 and 39 m, respectively. This was done by calculating the advance and recession rates at the 4 locations in each plot in metres per minute (metres from the top edge of the plot, m min^−1^). The normalized advance and recession times at each location were then calculated by multiplying the rates by the distance of the location from the top of a 40 m long plot.

#### 2.4.4 Irrigation amount

The amount of irrigation applied to each plot was determined for all irrigations except the first three using a Stingray® 2.0 Portable Level-Velocity Logger flow metre placed in the inlet pipe to each plot. For the first three irrigations of the first crop, the amount of irrigation water was computed from the duration of the irrigation and the flow rate of the tubewell (assuming conveyance losses negligible as the pump was less than 10 m away). Each year the rice replicates received 13–18 irrigations. It was not possible to complete the irrigation of all three replicates (farmers’ fields) on the one day. Therefore the number of irrigations of rice replicates varied as it sometimes rained after 1 or 2 replicates had been irrigated, and remaining replicates did not need irrigation. The 2014 maize crop was not irrigated as rainfall was adequate to maintain soil water tension above the threshold for irrigation throughout the season, but the 2015 crop received 2 post-sowing irrigations. All wheat crops received 5 irrigations except for the 2015–16 crop in the RW system which received 4 irrigations (Table S2). Irrigation water productivity (WPi) was computed as kg grain m^−3^ of water applied.

#### 2.4.5 Volumetric soil water content

Volumetric soil water content (VWC) was determined near the centre of the head, middle, and tail sections of every lasered plot by a PR2 Profile probe®. Measurements were made at 10, 20, 30, 40, 60 and 100 cm soil depth in tubes installed mid-way between the plant rows in the flat plots, and in the centre of the beds with soil depth taken from the tops of the beds. A total of 93 days of readings in rice (63 in 2014 and 30 in 2015), 54 days in wheat (29 in 2014–15 and 25 in 2015–16), and 72 days in maize (59 in 2014 and 13 in 2015) were taken. In both years in all crops, readings were made 1–2 h before irrigation (BI) and 1–2 days after irrigation (AI) (as soon as the plots could be accessed without damaging the soil/crop), and otherwise approximately twice weekly.

### 2.5 Economic analysis

Gross return, total cost of production (excluding land rent), and gross margin for rice, wheat, and maize crops were determined for each treatment each year. The costs of production considered were total labour (hired plus family labour), inputs (seed, fertilizer, herbicide), and machinery hire (for tillage, land levelling, bed formation, rice transplanting, threshing, winnowing). The cost of laser land levelling for each gradient and for bed formation were based on the time taken for each plot and converted into h ha^−1^, and custom hire charges for these operations. The cost of custom hire laser land levelling was used for 0% gradient, and the cost for 0.1% gradient was calculated from this by multiplying by the proportionate change in time to achieve 0.1% gradient (Tables S5 and S6). The cost of laser land levelling was assigned to the first crop (rice, maize) each year. The cost for irrigation water was computed for each treatment from the duration (hours) of each irrigation in each plot and the current rental charge of 100 INR h^−1^ for the 6.5 hp motorized pump. The labour requirement and costs of inputs and machinery hire for each crop were based on the findings of recall surveys of participating farmers, key informant surveys of agricultural input supplier, and machinery service providers. Government fixed prices for grain and straw were used as the price of outputs. Gross margin was computed from the difference between gross return and total cost of production. Costs were converted to USD using an exchange rate of 62 INR = 1 USD. It is likely that the determination of gross margin is conservative because farmers supply their own labour, in addition to hiring labour.

### 2.6 Statistical analysis

Analysis of variance (ANOVA) with a general linear model (GLM) by segregating the sampling error from the experimental error was conducted using the ‘Agricolae’ package of R Version 3.6.1. Analysis of variance for a randomized complete block design was used to compare the effects of treatment (three in RW, five in MW), year (and section in the case of yield) and their interactions on grain yield, irrigation amount and WPi for each crop within each system. For the MW system, ANOVA was also used to check for interactions between land gradient, configuration and section each year. None of the interactions were significant, apart from a significant interaction between gradient and section on grain yield of wheat in the MW system in 2014–2015. Therefore, the year and treatment means for grain yield, irrigation amount and WPi, plus the section means for grain yield, are presented (Table 1, Table 2, Table 4, Table 5). The effects of treatment on the water advance, recession, intake-opportunity, and VWC were determined using ANOVA for each sampling/repeated point and the differences between individual treatments were analyzed using Fisher’s Protected Least Significant Difference (LSD).

**Table 1:** Grain yield (t ha^−1^) of rice, maize, and wheat as affected by land gradient and configuration (establishment methods; flat and bed) under rice-wheat and maize-wheat cropping systems in Vaishali, Bihar, during 2014–2016. Values followed by the same letter within the same factor and column are not significantly different.

Treatment	Rice-wheat system		Maize-wheat system	
	Rice	Wheat	Maize	Wheat
Farmer levelling (FL)	4.74^b^	4.01^b^	5.41^c^	4.34^c^
0% Flat	4.97^b^	4.31^ab^	5.72^bc^	4.65^bc^
0.1% Flat	5.46^a^	4.57^a^	5.96^ab^	4.84^ab^
0% Bed	–	–	6.21^a^	4.97^ab^
0.1% Bed	–	–	6.36^a^	5.18^a^
Treatment LSD at p = 0.05	0.35	0.34	0.40	0.34
*Year*				
2014–15	5.74^a^	4.42	6.32^a^	5.14^a^
2015–16	4.37^b^	4.17	5.54^b^	4.45^b^
*Section*				
Head	5.17	4.33	6.18	4.75
Middle	4.98	4.28	5.88	4.76
Tail	5.02	4.28	5.73	4.87
*ANOVA (p values)*^*1*^				
Year	***	ns	***	***
Treatment	***	**	*	***
Section	ns	ns	ns	ns
Year x treatment	ns	ns	ns	ns
Treatment x section	ns	ns	ns	ns
Year x treatment x section	ns	ns	ns	ns

^1^* denotes p < 0.05, ** denotes p < 0.01, *** denotes p < 0.001; ns = not significant.

**Table 2:** Wheat yield as affected by the interaction between gradient and section in the MW system in 2014–15.

**Gradient**	**Section**		
	**Head**	**Middle**	**Tail**
0%	5.40	5.18	4.99
0.1%	5.10	5.26	5.88
LSD at p = 0.05	0.34

**Table 3:** Mean irrigation water intake opportunity time (min) as affected by land gradient and configuration in rice, wheat, and maize crops under RW and MW systems. Data are the average over all irrigations of maize (2015 only) and of wheat and rice over two years.

Treatment	Rice-wheat system		Maize-wheat system	
	Rice	Wheat	Maize	Wheat
Farmer levelling (FL)	410	278	210	200
0% Flat	309	240	232	276
0.1% Flat	403	260	187	240
0% Bed	–	–	272	346
0.1% Bed	–	–	280	297
	**	ns	***	***
LSD at p = 0.05	66.7	39.8	43.5	38.8

^1^* denotes p < 0.05, ** denotes p < 0.01, *** denotes p < 0.001; ns = not significant.

**Table 4:** Irrigation amount (mm) as affected by land gradient and configuration under RW and MW cropping systems in Vaishali, Bihar, 2014–2016. Values followed by the same letter within the same factor and column are not-significantly different.

Treatment	Rice-wheat system		Maize-wheat system	
	Rice	Wheat	Maize (2015 only)	Wheat
Farmer practice (FP)	1241^a^	304a	354^a^	275^a^
0% Flat	928^b^	233b	206^b^	251^ab^
0.1% Flat	842^b^	202b	183^c^	230^ab^
0% Bed	–	–	163^d^	219^b^
0.1% Bed	–	–	149^e^	208^b^
Treatment LSD at p = 0.05	245	49	13	46
*Year*				
2014–15	1200^a^	268^a^	–	292^a^
2015–16	807^b^	224^b^	191	181^b^
*ANOVA (p values)*				
Year	**	*	–	***
Treatment	*	**	***	*
Year x treatment	ns	ns	–	ns

^1^* denotes p < 0.05, ** denotes p < 0.01, *** denotes p < 0.001; ns = not significant.

**Table 5:** Irrigation water productivity (WPi, kg m^−3^) as affected by land gradient and configuration under RW and MW cropping systems in Vaishali, Bihar, India. Values followed by the same letter within the same factor and column are not-significantly different.

Treatment	Rice-wheat system		Maize-wheat system	
	Rice	Wheat	Maize (2015 only)	Wheat
Farmer practice (FP)	0.42^b^	1.37^c^	1.95^d^	1.72^c^
0% Flat	0.60^a^	1.93^b^	2.74 ^cd^	2.1^bc^
0.1% Flat	0.71^a^	2.32^a^	3.07^bc^	2.50^ab^
0% Bed	–	–	3.67^ab^	2.66^ab^
0.1% Bed	–	–	4.14^a^	2.93^a^
Treatment LSD at p = 0.05	0.12	0.32	0.78	0.68
*Year*				
2014–15	0.58	1.93	–	2.95^a^
2015–16	0.57	1.82	3.11	1.82^b^
*ANOVA (p values)*				
Year	ns	ns	–	***
Treatment	**	***	***	**
Year x treatment	ns	ns	–	ns

^1^* denotes p < 0.05, ** denotes p < 0.01, *** denotes p < 0.001; ns = not significant.

## 3 Results

### 3.1 Weather

**Fig. 3:**
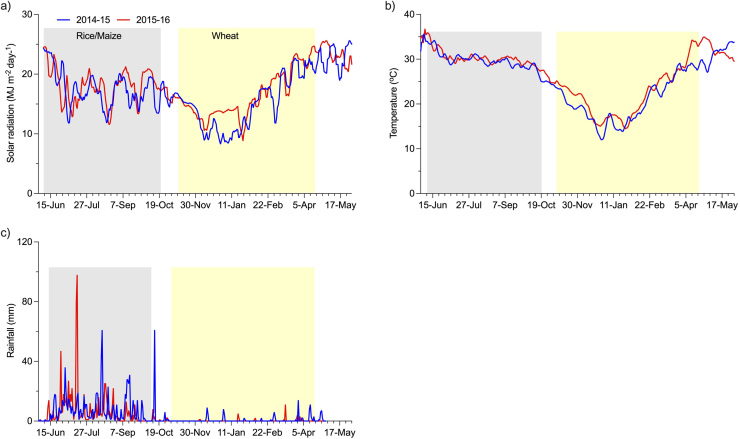
Moving 7-day average solar radiation, temperature and daily rainfall in 2014–15 and 2015–16.

Solar radiation was higher in 2015–16 than 2014–15 during most of the reproductive and grain filling period for rice and maize (Sep-Oct). For wheat, solar radiation in 2016 was higher than in 2015 during the vegetative (Nov-Dec) and the reproductive and grain filling period (Feb-Mar) (Fig. 3a). In the rice/maize season (June-October), average temperature was about 0.5 °C higher in 2015 than in 2014 (Fig. 3b). For wheat (Nov-April), the average temperature was 2.1 °C higher in 2015–16 than 2014–15, with the biggest difference (4.2 °C in April) towards the end of the grain filling period. Total rainfall was 1176 mm and 969 mm in 2014–15 and 2015–16, respectively, with about 90% falling during the rice/maize season each year (Fig. 3c). Although total rainfall was higher in the first year, the amount of rainfall received in July 2015 (285 mm), soon after establishment of rice and maize, was 49% higher in 2015 than in July 2014.

### 3.2 Grain yield of rice, maize and wheat as affected by gradient and configuration

In the both the RW and MW systems, there was a consistent trend for higher yield in the 0% flat treatments than in FL in all crops, but the differences were never significant (Table 1). Within land configuration (flats or beds), there was no significant effect of gradient on the yield of wheat (both systems) or maize, but yield of rice with 0.1% gradient was significantly higher (by 10%) than yield with 0% gradient. Yield of maize and wheat on beds was higher than yield on the flat (by 0.44 t ha^−1^ or 8% in maize and 0.32 t ha^−1^ or 7% in wheat). For maize, there was a general trend for yield to decline with distance from the top of the plots each year (Fig. S2), but the differences were never significant (Table S3). There were no consistent trends for the effect of section on yield of wheat in either system. However, in 2014–15, there was a significant interaction between gradient and section on yield of wheat in the MW system (Table 2). With 0% gradient, yield declined significantly between the head and tail sections, whereas with 0.1% gradient, yield in the tail section was significantly higher than in the head section.

There was a consistent trend for higher yields of all crops in the first year than the second year (Table 1). Rice yield in 2014 was higher by 23% (1.3 t ha^−1^) than in 2015, maize yield by 14% (0.90 t ha^−1^), and wheat yield by 13% (0.69 t ha^−1^) and 7% (0.33 t ha^−1^, not significant) in the MW and RW systems, respectively.

### 3.3 Advance, recession and intake opportunity time in rice-wheat and maize-wheat systems

#### 3.3.1 Water advance

**Fig. 4:**
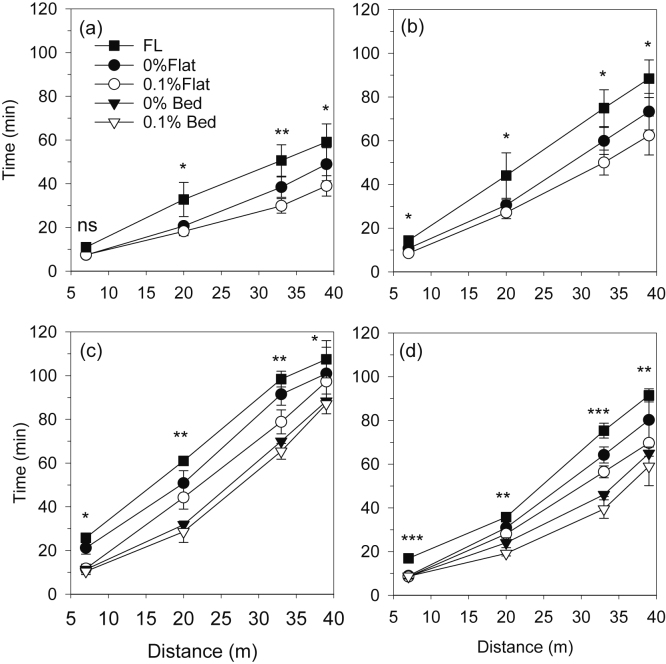
Time for the irrigation water to advance to the middle of each section in a 40 m long plot, as affected by land gradient in rice (a) and wheat (b) under the rice-wheat system and by land gradient and configuration in maize (c) and wheat (d) in the maize-wheat system. Data are the average over all irrigations of maize and wheat over two years, and 7 irrigations of rice each year. Vertical bars are standard errors. Note: ns: nonsignificant (p > 0.05). *: significant at p < 0.05; **: significant at p < 0.01; ***: significant at p < 0.001.

For the RW system, the average time for the irrigation water to reach the end of the rice plots (40 m) was 39 min for 0.1%, 49 min for 0% and 59 min for FL (Fig. 4a). The water advance time was thus decreased by 17% and 34% with 0% and 0.1% land gradient, respectively, compared to FL. Similarly, in wheat, the advance time was decreased by 17% and 30% in 0% and 0.1% gradients compared with FL (Fig. 4b). The faster advance in rice than wheat within respective treatments was probably due to both the higher flow rates used for rice and wetter soil (lower irrigation threshold) at the time of irrigation.

In the MW system, the time for water to reach the end of the plots ranged from 85 to 105 min. The advance was faster in bed than flat plots, regardless of gradient, and slowest in FL (Fig. 4c,d). In both the maize and wheat crops on the beds, there was no significant difference in advance time between 0% and 0.1% gradients throughout the length of the plots. However, on the flat, the advance was faster with 0.1% than 0% gradient in the tail section, and in either the middle section (maize) or at the end of the plots (wheat). As for the RW system, the advance was slower in FL than any other treatment combination.

#### 3.3.2 Water recession

**Fig. 5:**
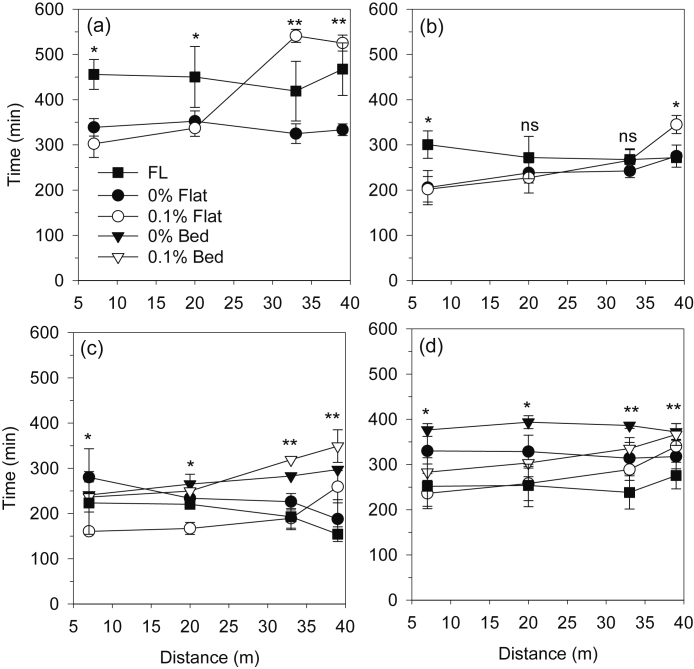
Time for the irrigation water to recede from the middle of each section in a 40 m long plot, as affected by land gradient and configuration in rice (a) and wheat (b) under the rice-wheat system, and in maize (c) and wheat (d) under maize-wheat system fields. Data are the average over all irrigations of maize and wheat over two years, and 7 irrigations of rice each year. Vertical bars are standard errors. Note: ns: nonsignificant (p > 0.05). *: significant at p < 0.05; **: significant at p < 0.01; ***: significant at p < 0.001.

The recession time was longer in rice than the non-rice crops, and ranged from about 300–550 min across treatments and plot sections in rice (Fig. 5). In both the RW and MW systems, the recession time for wheat ranged from about 200–400 min, while for maize it ranged from about 150–350 min. There were few clear treatment trends in recession time. In the 0.1% flat plots, the water recession time tended to increase towards the end of the plots. In the MW system, recession times in wheat were shorter in the 0.1% beds than in the 0% beds, however, in maize recession times were longer in the 0.1% beds in the tail and end of the plots. Within the same gradient, water recession times were generally shorter in flat plots than beds.

#### 3.3.3 Intake opportunity time

**Fig. 6:**
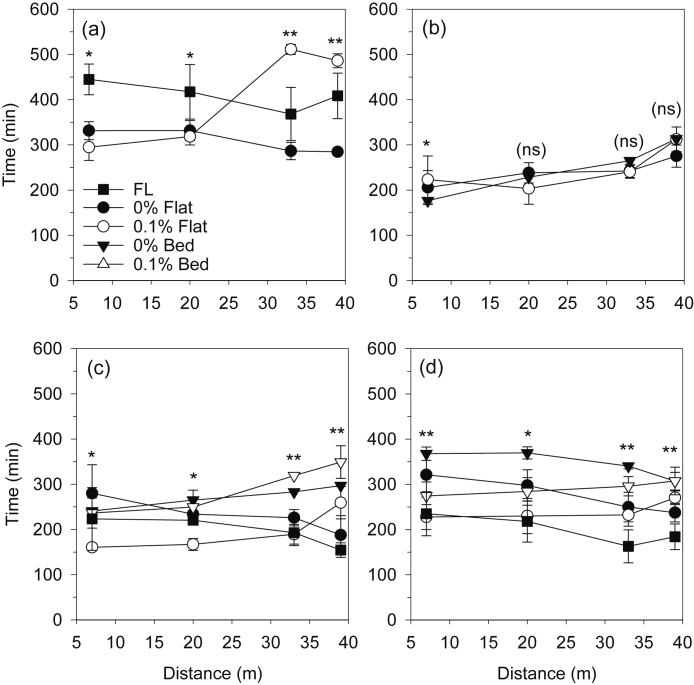
Intake opportunity time (min) for the irrigation water from the middle of each section in a 40 m long plot, as affected by land gradient and configuration in rice (a) and wheat (b) under the rice-wheat system, and in maize (c) and wheat (d) under maize-wheat system fields. Data are the average over all irrigations of maize and wheat over two years (maize 2015 only), and 7 irrigations of rice each year. Vertical bars are standard errors. Note: ns: nonsignificant (p > 0.05). *: significant at p < 0.05; **: significant at p < 0.01; ***: significant at p < 0.001.

Trends in intake opportunity time were generally similar to trends in recession time because recession times were much longer than advance times (Fig. 6). Mean section intake opportunity times ranged from 300 to 500 min in rice, 100–300 min in maize, 200–300 min in wheat in the RW system, and 200–375 min in wheat in the MW system. In rice, mean intake opportunity time in 0.1% flat and FL was similar (~400 min) and significantly higher (by 30%) than in 0% flat (Table 3). In the RW system, there was no effect of levelling treatment on mean intake opportunity time in wheat, but in the MW system, intake opportunity time in FL was significantly lower than in all other treatments. In the MW system, intake opportunity time was increased on the beds in comparison with flats. There was a trend for 0.1% gradient on the flat to reduce intake opportunity time in comparison with 0% in both crops, and in wheat on beds. However, the gradient did not affect intake opportunity time for maize on beds.

### 3.4 Irrigation amount and irrigation water productivity

#### 3.4.1 Irrigation amount

Land gradient and configuration significantly affected irrigation amount in all crops (Table 4). In the RW system, 0% gradient on the flat reduced the amount in both rice and wheat by about 25% compared with FL, although the magnitude of the reduction was much larger in rice (313 mm) than wheat (71 mm). Changing to 0.1% gradient did not further reduce irrigation amount to either crop. In the MW system, there was no significant interaction between gradient and configuration (Table S4). The flat treatment with 0% gradient reduced irrigation amount in maize by 42% (148 mm) compared with FL, and a 0.1% gradient further reduced irrigation amount by 23 mm. While there was also a declining trend with laser levelling and increased gradient, irrigation amount to wheat was similar in FL and 0% and 0.1% flat plots. Beds further decreased irrigation amount to maize, with reductions of 54% and 58% with 0% and 0.1% gradient, respectively, compared with FL. Irrigation amount to wheat on beds was reduced by about 25% in comparison with FL, and was not affected by gradient. Irrigation amount to rice and wheat was significantly lower in 2014–5 than in 2015–6 (Table 4), while maize did not require irrigation in the first year.

#### 3.4.2 Irrigation water productivity

Irrigation water productivity was significantly lower in FL than in all gradient x configuration treatment combinations in all crops, except for similar values on 0% flat in the MW system (Table 5). Within land configuration, there was a trend for higher WPi with 0.1% than 0% gradient, but the difference was only significant for wheat on the flat in the RW system. In the MW system, there was no significant interaction between gradient and configuration (Table S4). WPi of maize was significantly higher (by 34%) on beds than flats, and by 21% in wheat.

Irrigation water productivity of both crops in the RW system was similar each year. However, in the MW system, WPi of wheat was higher in 2015 than in 2014.

### 3.5 Soil water content in rice-wheat and maize-wheat systems

**Fig. 7:**
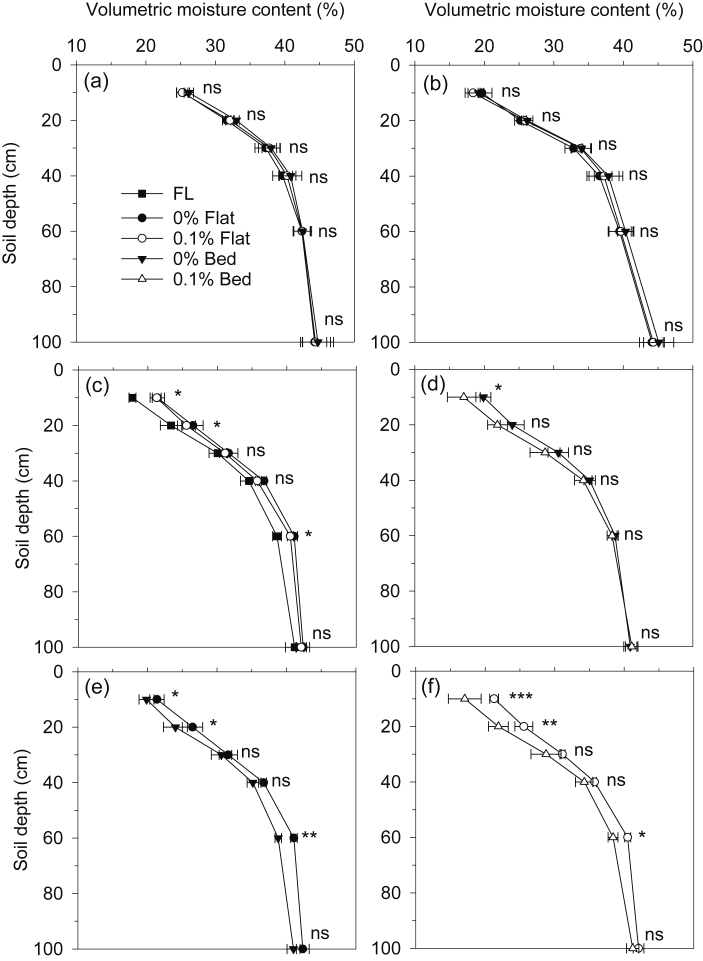
Average volumetric soil water content profiles of each treatment in rice (a) and wheat (b) crops in the RW system; and in FP, 0% and 0.1% flat (c); 0% and 0.1% beds (d); 0% flat and beds (e), and 0.1% flat and beds (f) in maize-wheat system during 2014–2016 in Vaishali, Bihar, India. Figures c-f are from the average of maize and wheat crops of maize-wheat system. Data from an average of two years from all sampling dates. Horizontal bars are standard errors. Note: ns: nonsignificant (p > 0.05). *: significant at p < 0.05; **: significant at p < 0.01; ***: significant at p < 0.001.

Volumetric soil water content varied during the crop growing period to a depth of about 30 cm during rice, 100 cm during maize, and 80 cm during wheat (Figs. S3 and S4). Average VWC content (averaged over all measurements over both years) was similar within depth in all treatments in the RW system, within each crop (Fig. 7a,b). In the MW system, average VWC in the topsoil (at 10 and 20 cm) of FL was lower than in the other treatments (Fig. 7c). A 0.1% gradient in the beds had almost no effect on average VWC in wheat, except for a lower value at 10 cm (Fig. 7d). In maize, VWC of the topsoil (10 and 20 cm) and at 60 cm was lower on beds than flats with the same gradient.

### 3.6 Economic analysis

With farmer levelling (FL), the average (over two years) total cost of rice production was USD 1167 ha^−1^, more than double that of maize production, and roughly double the cost of wheat production in each system (Table 6). The higher production cost of rice was mainly due to much higher irrigation cost, which was 53% of the total rice production cost (Tables S5−S7). Laser levelling slightly decreased the cost of production of rice over FL due to reduced irrigation cost more than offsetting the increased levelling cost. Laser levelling and beds increased the production cost of maize by USD 150–210 ha^−1^ and decreased the production cost of wheat slightly.

**Table 6:** Variable cost, gross return and gross margin for rice, wheat, maize crops production under rice-wheat and maize-wheat systems as affected by land gradient and configuration.

Land configuration and gradient	**Rice-wheat system**						
	Rice			Wheat			System
	Gross return (USD ha^-1^)	Total cost production cost (USD ha^-1^)^1^	Gross margin (USD ha^-1^)	Gross return (USD ha^-1^)	Total production cost (USD ha^-1^)^1^	Gross margin (USD ha^-1^)	Gross margin (USD ha^-1^)
Farmer levelling (FL)	1149^b^	1167	-18^c^	998^b^	642	356^c^	338^c^
0% Flat	1204^b^	1083	121^b^	1072^a^	599	474^b^	595^b^
0.1% Flat	1322^a^	1099	223^a^	1135^a^	583	552^a^	775^a^
*Year*							
2014	1382^a^	1068	314^a^	1082	627	456	769^a^
2015	1067^b^	1164	-96^b^	1055	589	466	370^b^
*Section*							
Head	1253	1116	137^a^	1076	608	468	605
Middle	1206	1116	90^b^	1065	608	457	547
Tail	1215	1116	99^b^	1065	608	457	556
	**Maize-wheat system**						
	Maize			Wheat			System
Farmer levelling (FL)	1310^c^	457	853^b^	1083^c^	592	491^c^	1344^d^
0% Flat	1384^bc^	611	774^b^	1160^bc^	576	584 ^bc^	1358^d^
0.1% Flat	1443^ab^	645	798^ab^	1206^ab^	572	635^ab^	1433^c^
0% Bed	1504^a^	623	881^a^	1238^ab^	563	675^ab^	1556^ab^
0.1% Bed	1540^a^	663	877^a^	1291^a^	557	734^a^	1612^a^
*Year*							
2014	1513^a^	518	995^a^	1274^a^	616	658^a^	1653^a^
2015	1360^b^	564	796^b^	1117^b^	527	590^b^	1386^b^
*Section*							
Head	1496^a^	541	955^a^	1184	572	613	1567
Middle	1425^a^	541	884^ab^	1188	572	616	1500
Tail	1388^b^	541	848^b^	1215	572	643	1490

^1^For the detail of total variable costs, please see supplementary tables (Tables S4−S6).

The returns from maize were higher than from all other crops, within treatment; similarly, the returns from rice were higher than those from wheat, more so in the RW system (Table 6). Laser levelling with 0% gradient and flat configuration gave similar returns to FL in all crops except for wheat in the RW system, which had higher returns in the lasered treatment. Laser levelling with 0.1% gradient and flat configuration significantly increased returns from all crops in comparison with FL. Beds with either gradient further increased returns of both maize and wheat relative to FL.

Rice was by far the least profitable crop; FL rice had a small negative gross margin, while rice with laser levelling gave relatively small gross margins (USD 121–223 ha^−1^) (Table 6). Maize was the most profitable crop, with gross margins ranging from USD 770–880 ha^−1^. Wheat was also profitable with gross margins ranging from USD 560–640 ha^−1^.

Using FL, gross margin of the annual MW system was 4 times that of the RW system (Table 6). In the lasered treatments with flat configuration, gross margin of the MW system was 2–3 times that of the RW system. A 0% gradient with flat configuration significantly increased gross margin of the RW system over FL, but not the MW system. A 0.1% gradient on the flat significantly increased the gross margin of both systems above systems with 0% gradient. The use of beds in the MW system further increased gross margin over the flat treatments, with no effect of slope.

## 4 Discussion

### 4.1 Comparison of farmer practice levelling (FL) and laser levelling with 0% gradient and flat configuration

The lower irrigation amounts and higher WPi of the 0% lasered flat treatments in comparison with FL are consistent with the findings of other studies in small farmers’ fields in South Asia (Jat et al., 2006, Jat et al., 2015, Aryal et al., 2015). These studies also often show yield benefits when comparing 0% laser levelling on the flat with farmer practice. The lack of a significant effect on yield of any crop in our study is probably because the same (best) management practices were used in both the lasered and FL treatments in our study. The only differences between FL and the 0% laser levelled treatments in our study were the use of farmer levelling methods. Farmers’ practice in other studies often involves a range of typical (sub-optimal) farmer management practices other than levelling. Rice grown with FL was not profitable (slightly negative gross margin) because of the high irrigation cost, but laser levelling gave a small profit. In contrast, laser levelling with 0% gradient and flat configuration did not increase the profitability of maize because the reduction in irrigation cost was too small to offset the cost of laser levelling.

### 4.2 Effect of gradient (0.0% versus 0.1%)

#### 4.2.1 Irrigation advance and amount

Laser levelling with a 0.1% gradient increased the advance rate of the irrigation water along the flat plots in all crops in both cropping systems, as hypothesized. The time taken for the water to reach the end of the plots was decreased by 9 min or 12% in 0.1% flat treatments compared with 0%. While there was also a trend for more rapid advance in the beds with 0.1% than 0% gradient, the effect was very small. The more rapid advance with 0.1% gradient resulted in a consistent trend for lower irrigation amount than with 0% gradient, but the difference was never significant, except for maize grown on flats and on beds. Plot size was probably too small for the effect of gradient on irrigation amount to be significant on the clay loam soil.

The potential for a small gradient to reduce irrigation amount increases with plot size and on more permeable soils (González et al., 2011). On a highly permeable clay loam soil in the Philippines, a 0.1% gradient resulted in a 22% irrigation reduction for dry seeded rice in comparison with 0% gradient (Evangelista, 2019). The latter study was conducted in 50 m x 32 m plots and used the same irrigation scheduling rules as our study. In a simulation study using the WinSRFR and POZAL models to optimize basin irrigation design, González et al. (2011) found a 20% reduction in irrigation amount for rice with a gradient of 0.04% in comparison with 0% gradient in 200 m x 50 m plots with a flow rate of 100 l s^−1^. In a similar study in Brazil, Winkler et al. (2018) compared 0%, 0.20%, 0.25%, 0.28% and 0.40% gradients on the flat for rice. They recommended using >0.1% gradient to improve drainage and soil drying, and thus ability to establish the next (upland) crop after rice harvest.

In addition to gradient, the water advance rate depends on several other factors including the discharge rate of the irrigation pump, surface roughness, infiltration rate, and plot geometry (Walker et al., 2006, Bautista et al., 2009). A higher discharge rate increases the water advance rate and reduces irrigation time, thus reducing deep drainage losses and irrigation amount (Smith et al., 2005). Similarly, optimizing the cut-off time for irrigation can increase irrigation water productivity (Smith and Uddin, 2020). Further experimental and simulation studies are needed to optimize the system for farmers’ fields in the IGP. Such studies should consider management factors which farmers can manipulate, including gradient, plot geometry, flow rate, cut-off time, and the use of beds. Variation in seasonal conditions, especially rainfall, also needs to be considered.

#### 4.2.2 Grain yield

Laser levelling with a 0.1% gradient increased rice yield by 10% (0.5 t ha^−1^) in comparison with 0% gradient. This was an unexpected result which is difficult to explain, and further studies are needed to validate this finding. A similar study done on a highly permeable clay loam soil of Philippines reported no effect of gradient (0% vs 0.1%) on grain yield (Evangelista, 2019). The average intake opportunity time in our study was about 30% longer with 0.1% gradient compared with 0%, suggesting that the plots were waterlogged for longer after each irrigation, which could have been beneficial to the rice crop. But the increased intake opportunity time only occurred in the tail section of the plots (Fig. 6), whereas yields in all three sections were similar. There was a consistent trend for higher yields of maize and wheat with 0.1% gradient compared with 0%, within both bed and flat configurations, but the differences were never significant. In wheat and maize on the flat, and in wheat on beds, intake opportunity time decreased with 0.1% gradient compared with 0% gradient, suggesting the possibility of reduced irrigation-induced waterlogging and leaching.

The higher wheat yield in the tail end of the 0.1% treatment in the MW system could have been due to higher water availability as a result of the longer intake opportunity time in this section (Fig. 5D). Average soil volumetric water content of the top 30 cm during the crop growing period was significantly higher (by 10%) in the tail end (25.9 cm^3^ cm^−3^) than the head end (23.7 cm^3^ cm^−3^). In contrast, with 0% gradient, wheat yield was higher in the head section than the tail section. This was also associated with higher intake opportunity time in the head section, and higher average soil water content.

The higher wheat yields in the first year than the second year were probably due to the much later sowing (Table S2) leading to later flowering (10–20 Feb 2015, 20 Feb-1 Mar 2016) pushing grain filling inter warmer weather. Added to that, temperatures were higher in the latter part of the second wheat season than the first season (Fig. 3). The yield difference across years was much greater for wheat in the MW system than the RW system. This is likely due to a synergy in the first year between the cooler grain filling period and more favourable soil conditions in the MW system. While the soil was not puddled for rice, it remained at or near saturation throughout the season. Prolonged periods of waterlogging can affect the availability of nutrients in subsequent upland crops, especially phosphorus (Willett, 1979, Willett and Higgins, 1978). Waterlogging can also lead to soil structural decline and adverse effects on upland crops (Bazzoffi and Nieddu, 2011).

#### 4.2.3 Irrigation water productivity (WPi)

There was a consistent trend for higher WPi with 0.1% than 0% gradient (within the same configuration) in all crops in both systems, but the differences were not significant except for wheat in the RW system. In the latter case, WPi was increased by 23% or 0.42 kg m^−3^.

In the MW system, the effect of gradient on WPi was never significant due to the lack of significant or substantial effects on both irrigation amount and yield.

#### 4.2.4 Overall benefits 0.1% versus 0% gradient

For the RW system, the results suggest no to marginal benefits in irrigation amount and WPi from laser levelling with a 0.1% gradient in comparison with 0% gradient in farmers’ irrigation plots of the size (~15 m x ~40 m) typical of those found in the study region, and indeed in much of Bihar, Eastern India. In the RW system, by far the bigger biophysical gains were from changing from farmer levelling practice to laser levelling with 0% gradient on the flat. Those gains came in the form of substantial reductions in irrigation amount which led to greatly increased WPi in both crops (by ~40%), while yield was not affected. The saving in irrigation cost with laser levelling with 0% gradient more than offset the cost of levelling, resulting in a small profit for rice, and also increased the profitability of wheat and the RW system. The yield increase with 0.1% gradient over 0% and FL further increased gross margin for both rice and the total RW system with 0.1% gradient. This rice yield increase warrants further investigation given the large and increasing RW area currently being laser levelled with 0% gradient.

As for the RW system, laser levelling to 0% with a flat configuration significantly increased WPi of both crops in the MW system in comparison with FL, but by a lesser proportion (17% increase for both crops). This was due to trends for higher yield and lower irrigation amount, the latter small but significant in the case of maize. While there were trends for higher yield and lower irrigation amount (the latter significant in the case of maize) in changing from 0% to 0.1% gradient, this did not lead to a significant increase in WPi. The gains in WPi were much bigger when changing from FL to beds, regardless of gradient, and slightly more so in beds with a 0.1% gradient, in comparison with FL (Section 4.2, below). Whether laser levelling prior to bed formation confers additional benefits in comparison with farmer levelling prior to bed formation cannot be ascertained from this study. Laser levelling with a small gradient is common practice for irrigated crops grown on beds in large fields in developed countries, to reduce irrigation time and deep drainage losses and facilitate surface drainage. Lasering with a 0.1% gradient and flat configuration did not increase gross margin of maize or wheat in comparison with 0% gradient, but it did significantly increase gross margin of the MW system. This demonstrates the importance of considering the total system rather than individual crops alone. We repeated laser levelling each year to ensure that the desired gradients were maintained, as our primary focus was on the effect of gradient on irrigation amount and. However, lasering each year is probably not needed, especially when there is no puddling, and even less so if zero till is used to established all crops. In this case, profitability of the laser levelled and bed treatments would be considerably higher for the maize and rice crops, as the current analysis includes the cost of tillage prior to laser levelling, in addition to the cost of levelling itself (and bed formation).

### 4.3 Effect of raised beds on irrigation amount, yield and WPi in the maize-wheat system

**Fig. 8:**
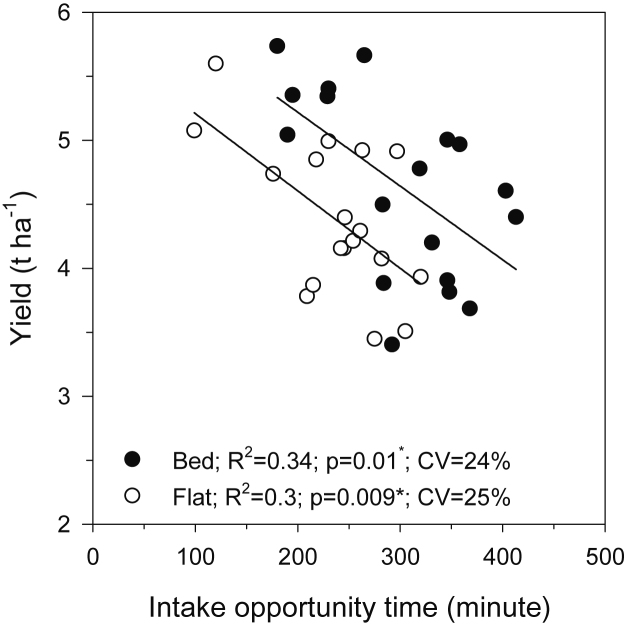
Relationship between yield and intake opportunity time in wheat in maize-wheat system 2015–16. Coefficient of variation (CV) is the measure of variability in intake opportunity time as affected by variability mostly among the replications (mainly the soil type between the fields). This is only for one system in one year, the relationships in other systems and years were not significant.

In laser levelled plots, raised beds significantly increased yield of maize by 8% (0.5 t ha^−1^), reduced irrigation amount by 20% (40 mm) and thus increased WPi by 34% (1.0 kg m^−3^) in comparison with the laser levelled flat plots, and the gains were even greater in comparison with FL. The results are consistent with the findings of other studies in the region which compared raised beds with farmers’ practice (Choudhary et al., 2018, Jat et al., 2019). While the same trends occurred in comparisons of wheat on laser levelled beds and flats, the differences were not significant (except for higher WPi of wheat on 0.1% beds compared with flats). The yield increases in maize on beds occurred in 2014, when no irrigation was required, as well as in 2015 (2 post-sowing irrigations). There could be several reasons for the higher yield of maize (but not wheat) on beds. Bed formation involves shifting of topsoil from the furrows to the bed tops, and the maize was planted in the middle of the beds. Thus, the maize could have benefited from increased nutrient availability in the beds. On the other hand, the wheat was planted across the beds (topsoil enriched) and furrows (topsoil depleted), with no potential benefit to average yield across the plots and benefitted from the increased water availability in the tail section. Reduction in waterlogging is another potential benefit of beds (Walker et al., 2006, Bautista et al., 2009), especially on clayey soils such as in Vaishali. The duration of irrigation-induced waterlogging is influenced by intake opportunity time. Regression of intake opportunity time against maize yield in 2015 and wheat yield in 2014–15 showed no significant relationship between intake opportunity time and yield on either flats or beds (data not presented), suggesting no significant benefits of beds in terms of irrigation-induced waterlogging in these crops/years. However, in 2015–16 wheat, there was a significant decline in yield with intake opportunity time on both flats and beds (Fig. 8). The rate of decline was similar for both configurations (235 kg ha^−1^ h^−1^), but the intercept was about 0.5 t ha^−1^ higher on beds than flats. The similar rate of decline suggests that irrigation-induced waterlogging affected yield in that year, but to the same degree on both flats and beds. Why that may have been the case in only one crop/year/system is unclear. Waterlogging affects physiological processes, growth and yield of many annual crops and the yield reduction in annual crops ranges from 15% to 20% (Herzog et al., 2016). Yield of winter wheat and barley declined by 25% under poorly drained conditions in Denmark (Jensen et al., 2021). Waterlogging can also have adverse effects on the yield of maize (Tian et al., 2020). However, our results do not suggest reduced irrigation-induced waterlogging in the maize in 2015. Other studies have also shown that bed planting of wheat and maize increased the water advance rate, reduced irrigation amount, and increased yield and WPi in comparison with farmers’ practice (Sayre and Moreno Ramos, 1997, Jat et al., 2011, Araya et al., 2012).

In the MW system, beds resulted in significant increases in gross margin of maize and wheat over flats across gradients, and in gross margin of the MW system regardless of gradient. This further increased the total MW system gross margin to 116–120% of that of FL.

### 4.4 Field consolidation

The small size of farmers’ plots is seen as one of the major constraints to the modernization of agriculture in India, including the adoption of mechanization (Chand, 2017). To address the challenge of small plot size, land consolidation and collective action have been promoted to farming communities in recent times. The “Small Farmer Large Field (SFLF)” model has been recently introduced in eastern India to enable small farmers to benefit from economies of scale by integrating their small plots into one large field (Mohanty et al., 2018). The model encourages consolidation of plots within the landholding of a farmer to facilitate the adoption of modern technologies. The SFLF concept gained immense popularity in Vietnam (Mohanty et al., 2017, Ba et al., 2019). With larger plots under the SFLF model, irrigations will take longer if supply flow rates are not increased proportionate to the increase in field size, with potential negative effects on yield and WPi from increased waterlogging and longer irrigation time. The study conducted by (Lybbert et al., 2013) in eastern Uttar Pradesh (a neighbouring state of Bihar with similar agricultural landscape including plot size), highlighted an interesting perspective on "willingness to pay" as the main driver for the adoption of laser land levelling rather than plot size. The smallest plot size in their study was ~800 m^2^ (compared with ~600 m^2^ in our study). Under the SFLF model, the introduction of a small (e.g. 0.1%) gradient may be more beneficial in bigger fields, provided that irrigation supply rates increase proportionately, and that drainage infrastructure allows adequate surface drainage from the field to prevent backup of water. However, the prevailing lack of drainage systems to take water away from farmers’ fields will likely diminish the benefits of slope in larger fields for upland crops unless this is also addressed.

### 4.5 Effect of cropping system on wheat yield

Wheat yield was 10% higher in the MW system than the RW system, despite the fact that site history was similar for both systems (puddled transplanted rice, conventional till wheat). This is consistent with the findings of several other studies comparing RW and MW systems where the soil was puddled prior to rice transplanting (Choudhary et al., 2018, Kumar et al., 2018). However, in our study, the soil was not puddled prior to rice transplanting, and both RW and MW fields had a long history of puddling for rice prior to commencement of the experiment. The reasons for the higher wheat yield in the MW system cannot be elucidated from our study, but could include the effects of the much wetter soil conditions in rice than maize in the RW system, affecting nutrient availability in the succeeding wheat crop, in particular phosphorus (Willett, 1982), and soil structure (Bazzoffi et al., 2011).

## 5 Conclusions

This study showed that laser levelling with a small (0.1%) gradient in farmers’ plots (~15 m x 40 m) produced a consistent trend for reduced irrigation amount in rice, wheat and maize crops, in comparison with 0% gradient. However, the effect was not significant, except for a very small (14 mm) but significant reduction in irrigation amount to maize on beds. There was also a consistent trend for higher grain yield and WPi of all crops with 0.1% gradient, but the increase was never significant, except for grain yield of rice. The latter is an unexpected result which needs to be validated/explained. The results suggest no to marginal biophysical benefits of laser levelling with a 0.1% gradient in the RW system in comparison with 0%. By far the bigger irrigation and WPi benefits in the RW system came from changing from farmer levelling practice to laser levelling with 0% gradient, as is currently practised. This led to substantial reductions in irrigation amount (by 25% in rice and 23% in wheat), increases in WPi (by 43% in rice and 41% in wheat), and increases in gross margin of both crops and the total RW system. Gross margin of the RW system was greatest with lasering with a 0.1% slope because of higher rice and wheat yields. In contrast to the RW system, there were only small gains from lasering to zero gradient on the flat in the MW system. By far the bigger gains were from changing to beds (regardless of gradient), in terms of increased grain yield (by 8% in maize and 7% in wheat), WPi (by 34% in maize and 22% in wheat), and gross margin (by 17–20%), and decreased irrigation amount (by 20% in maize and 11% in wheat). Therefore, the findings to date suggest that, with current farmer plot size, laser levelling with 0.1% gradient is probably the best option for RW fields, while beds are the best option for MW fields. Whether laser levelling of the fields prior to bed formation is beneficial for the MW system cannot be ascertained from our study, and needs to be investigated. While there was a consistent trend for higher yields and WPi, and lower irrigation amount, with beds on a 0.1% gradient in comparison with 0% (in both maize and wheat crops), the effect of gradient was never significant. If the current push for consolidation of farmers’ small plots into larger fields is realized, the benefits of laser levelling with a small gradient instead of zero gradient will need to be reconsidered as larger plots will increase irrigation times. However, to fully realize the benefits, supply flow rates will need to be increased proportionate to the increase in field size, and appropriate drainage infrastructure will be needed to minimize back up of water at the tail end of the fields.

## Declaration of Competing Interest

The authors declare that they have no known competing financial interests or personal relationships that could have appeared to influence the work reported in this paper.
