# Supplementary material for: Land gradient and configuration effects on yield, irrigation amount and irrigation water productivity in rice-wheat and maize-wheat cropping systems in Eastern India
Source: Agric Water Manag. 2021 Sep 1;255:107036. doi: 10.1016/j.agwat.2021.107036 (PMC8363937; doi:10.1016/j.agwat.2021.107036)
Supplement: Supplementary file 1 — Supplementary material [file mmc1.docx]

**Supplementary information (SI)**

Table S1. Soil physical and hydraulic properties of the top 30 cm at the experimental sites in Vaishali, Bihar, during 2014-2016. Values are mean and range (in parentheses) of the three RW and three MW sites.

| Soil depth (cm) | Particle size analysis (%) | | | Volumetric water content (%) | | | Texture |
| --- | --- | --- | --- | --- | --- | --- | --- |
|  | Sand | Silt | Clay | Permanent wilting point (%)^1^ | Field capacity (%) | Saturation |  |
| 0-15 | 38 (12-70) | 29 (8-46) | 33 (10-45) | 19 (13-26) | 32 (23-42) | 50 (45-53) | Clay loam, sandy clay loam, silty clay loam |
| 15-30 | 36 (12-66) | 29 (8-46) | 35 (24-46) | 20 (13-26) | 33 (24-42) | 50 (46-53) | Clay loam, sandy clay loam, silty clay loam |

^1^Permanent wilting point, field capacity and saturation were determined using pedotransfer function as provided by Saxton et (1986).

Table S2. Crop management practices in the gradient treatments in the rice-wheat and maize-wheat cropping systems during 2014-2016.

|  |  | Rice | | Maize | | Wheat (both rice-wheat and maize-wheat systems) | |
| --- | --- | --- | --- | --- | --- | --- | --- |
|  |  | 2014 | 2015 | 2014 | 2015 | 2014-15 | 2015-16 |
| 1 | Variety | Arize^®^-6444 | Arize^®^-6129 | DKC 9144 | Dekalb Double | HD-2967 | HD-2967 |
| 2 | Planting date | 15 June- nursery sowing  30 June- transplanting | 10 June- nursery sowing  12 July- transplanting | 9 July | 6 July | 11 November | 23 November |
| 3 | Harvesting date | 19-20 October | 27 October | 12-18 October | 10 October 2015 | 15-17 April 2015 | 16 April 2016 |
| 4 | Method of field preparation | Land levelling and gradient creation, allow weed to germinate, dry-shallow tillage using rotovator 3-4 days before transplanting, pre-transplanting irrigation, | | Land levelling and gradient creation, allow weed to germinate, pre-sowing irrigation if the soil is not wet, Mera-71 application in moist soil. | | Pre-sowing irrigation 3-4 days before sowing, non-selective herbicide (Mera-71) application after irrigation in moist soil, and direct drilling into the untilled soil | |
| 5 | Crop establishment | Non-puddled transplanting using rice transplanting machine | | Manual dibbling | | Zero tillage wheat using 11-row seed cum-fertilizer drill | |
| 6 | Row-row spacing, cm | 23.8 | | 67.5 cm for both bed and flat planting. Plant to plant distance 20 cm | | 18 | |
| 7 | Seed rate (kg ha^-1^) | 20 | | 25 | | 120 | |
| 8 | Fertilizer rate N:P_2_O_5_:K_2_O kg ha^-1^ and application method | 120:60:40.  All P and K fertilizers applied basal after field preparation and before transplanting.  From the remaining N fertilizer after DAP, 1^st^ 25% urea N at 15 days after transplanting (DAT).  2^nd^ 37.5% urea N at 30-35 DAT in 1-2 cm standing water.  3^rd^ 37.5% at 50-55 DAT in 2-3 cm standing water during evening time  In 2014, 75 g (33% ZnSO_4_) +225 g urea in 15 L water sprayed on 29^th^ and 60^th^ DAT.  Also, 20 kg ZnSO_4_ ha^-1^ applied on 30^th^ DAT in 1-2 cm standing water  In 2015, Zinc applied in basal with P and K fertilizer @ 20 kg ZnSO4 ha^-1^.  75 g (33% ZnSO4) +225 g urea in 15 L water sprayed on 30^th^ and 60^th^ DAT | | 150:60:80.  All P and K fertilizers applied at basal during sowing in-furrow opened along the line and covered by the soil.  From the remaining N fertilizer after DAP, 1^st^ 40% urea N fertilizer after first intercultural operation dibbled besides the plant at 20-25 DAS at knee-height stage  2^nd^ 60% urea N at 40-45 DAS when 5-10% tassel appeared | | 150:60:60  All amounts of P fertilizer applied at sowing.  From the remaining N fertilizer (after DAP),  1^st^ 50% urea N and all K broadcast topdressed at 25-30 DAS 2-3 days after first irrigation during the CRI stage.  2^nd^ 50% urea at the flowering stage at 60 DAS after 2-3 days after irrigation in saturated soil | |
| 9 | Weed control | **Pre-emergence:** 600 ml Pretilachlor per acre 15 ml per 15 L water applied 2^nd^ day after transplanting.  **Post-emergence:** Bispyribac (10% w/v Nominee gold^®^ or Adora^®^) + pyrazosulfuron ethyl 10% WP (sathi) mixed 80 ml Sathi+ 80 g Nominee/acre/9 tank application on 30-35 DAT (PI stage), and  1 spot hand weeding at 45-50^th^ DAT | | **Pre-plant** Mera-71 @ 120 L solution per acre applied 2 days before sowing  **Pre-emergence** atrazine 1-3 days after sowing in moist soil,  + 1 spot **hand weeding** at 25 days after sowing  + **thinning** in maize within 30-40 DAS | | **Pre-plant:** Mera-71 @ 15 ml @ 120 L solution per acre applied 3-7 days before sowing after pre-sowing irrigation mixed with urea @ 100 g per tank if weeds were not growing actively.  **Post-emergence:** Herbicide Total (Sulfosulfuron 75% + metsulfuron-methyl 5% WG) @ 120 L solution per acre at 40-45 DAS | |
| 11 | No. of irrigation and irrigation scheduling | 13-17 irrigations,  10 kPa at 15 cm soil depth | 13-18 irrigations  10 kPa at 15 cm soil depth |  | 2 irrigations.  50 kPa at 32.5 cm soil depth | 5 irrigations to wheat in both RW and MW system.  35 kPa at 32.5 cm soil depth | 4 irrigations to wheat in RW system and 5 for MW system  35 kPa at 32.5 cm soil depth |
| 12 | Insecticide, fungicide & rodenticide application | 1^st^ Cypermethrine 5% + Chloropyriphos @ 1.5 ml/l in on 9^th^ Sept to control Gundhi bug and stem borer in rice.  2^nd^ Sprayed fungicide Nativo-Tebuconazole 50% + 25% Trifloxystrobin @ 10 g/15 lit water for false smut in rice on 6^th^ Sept.  3^rd^ Rodenticide (Zinc sulphite) to protect from rat on 10^th^ September | Furadan/Folidol sprayed to protect from termite and other insects:  1^st^ 21 July,  2^nd^ 5 August,  3^rd^ 31 August,  4th 24 September | Folidol powder sprayed on14^th^ July & 29^th^ July.  Furadan granule on the tip of the plant applied on 3^rd^ and 20^th^ August to control termites.  Cypermethrine 5% + Chloropyriphos spray on 19^th^ July to control stem borer | Aldrin:  1^st^ 10^th^ July  2^nd^ Aldrin-14-15 July  3^rd^ Furadan from the maize tip on 2^nd^ Aug.  4^th^ Furadan/Folidol: 3-5 August,  28-29 August,  +  9-10 July- Chloropyriphos |  |  |

Table S3. Interaction between configuration (flats, beds), gradient (slope), and section (head, middle and tail) on grain yield in maize and wheat in the MW system.

| Configuration, gradient and sections | Maize grain yield (t ha^-1^) | | Wheat grain yield (t ha^-1^) | |
| --- | --- | --- | --- | --- |
|  | 2014-15 | 2015-16 | 2014-15 | 2015-16 |
| *Configuration (C)* |  |  |  |  |
| Flat | 6.19 | 5.50 | 5.11 | 4.38 |
| Bed | 6.65 | 5.92 | 5.49 | 4.65 |
| P value (C) | ns | ns | ns | ns |
| *Gradient (G)* |  |  |  |  |
| 0% | 6.30 | 5.63 | 5.19 | 4.43 |
| 0.1% | 6.54 | 5.79 | 5.41 | 4.60 |
| P value (G) | ns | ns | ns | ns |
| P value (C x G) | ns | ns | ns | ns |
| *Section (S)* |  |  |  |  |
| Head | 6.69 | 6.06 | 5.25 | 4.50 |
| Middle | 6.40 | 5.62 | 5.22 | 4.52 |
| Tail | 6.17 | 5.44 | 5.43 | 4.54 |
| Section (S) | ns | ns | ns | ns |
| S x G | ns | ns | ** | ns |
| S x C | ns | ns | ns | ns |
| S x G x C | ns | ns | ns | ns |
| Grand mean | 6.42 | 5.71 | 5.30 | 4.52 |

ns=non-significant; *= significant at <0.05; **= significant at <0.01 ; ***=significant at <0.001.

Table S4. Interaction between gradient (slope), configuration (flats, beds) on irrigation amount and irrigation water productivity (WPi) in maize and wheat in the MW system.

| Gradient and configuration | Irrigation amount  (mm) | | | Irrigation water productivity (WPi)  (kg grain m^-3^ irrigation water applied) | | |
| --- | --- | --- | --- | --- | --- | --- |
|  | Maize | Wheat | | Maize | Wheat | |
|  | 2015-16 | 2014-15 | 2015-16 | 2015-16 | 2014-15 | 2015-16 |
| *Gradient (G)* | | | | | | |
| 0% | 184 | 288 | 182 | 3.21 | 1.83 | 2.92 |
| 0.1% | 166 | 275 | 163 | 3.61 | 2.04 | 3.41 |
| *Configuration (C)* | | | | | | |
| Flat | 195 | 297 | 185 | 2.91 | 1.78 | 2.82 |
| Bed | 156 | 266 | 161 | 3.91 | 2.09 | 3.51 |
| *ANOVA* | | | | | | |
| Gradient (G) | *** | ns | * | * | ns | ns |
| Configuration (C) | *** | ns | ns | ** | ns | * |
| G x C | ns | ns | ns | ns | ns | ns |
| LSD at p=0.05 | 9.84 | - |  | 0.44 | - | 0.70 |
| Grand mean | 175 | 282 | 173 | 3.41 | 1.94 | 3.16 |

ns=non-significant; *= significant at <0.05; **= significant at <0.01 ; ***=significant at <0.001.

Table S5. Labor use and cost of production of rice in RW system during 2014-2015.

| Activity | Labor requirement (no. person-days ha^-1^) | | Input & machinery cost (USD ha^-1^) | | Total cost of each activity^9^ | |
| --- | --- | --- | --- | --- | --- | --- |
|  | 2014 | 2015 | 2014 | 2015 | 2014 | 2015 |
| Hybrid seed^1^ |  |  | 81 | 81 | 82 | 82 |
| Mat nursery | 8 | 8 | 29 | 29 | 55 | 55 |
| Land preparation^2^ |  |  | 44 | 44 | 44 | 44 |
| Transplanting (using Machine)^3^ | 4.2 | 4.2 | 40 | 40 | 54 | 54 |
| Fertilizer (Urea, DAP, Potash)^4^ | 3 | 3 | 104 | 104 | 114 | 114 |
| Weed control^5^ | 7 | 7 | 46 | 46 | 68 | 69 |
| Harvesting | 25 | 25 |  |  | 80 | 82 |
| Threshing^6^ |  |  | 32 | 32 | 32 | 32 |
| Drying and winnowing | 6 | 6 |  |  | 19 | 20 |
| *Laser levelling* |  |  |  |  |  |  |
| Land levelling in 0% |  |  | 68 | 68 | 68 | 68 |
| Land levelling in 0.1% |  |  | 45 | 45 | 45 | 45 |
| *Irrigation cost^7^* |  |  |  |  |  |  |
| Farmer levelling (FL) | 5 | 5 | 576 | 623 | 592 | 639 |
| 0% Flat | 5 | 5 | 395 | 500 | 411 | 517 |
| 0.1% Flat | 5 | 5 | 359 | 479 | 375 | 495 |
| Total no. of labor | 58 | 58 |  |  |  |  |
| *Total labor^8^, input and production costs* |  |  |  |  |  |  |
| Farmer levelling (FL) | 186 | 191 | 377 | 377 | 1141 | 1192 |
| 0% Flat | 186 | 191 | 445 | 445 | 1027 | 1138 |
| 0.1% Flat | 186 | 191 | 490 | 490 | 1037 | 1161 |

^1^Inputs costs in mat nursery includes the cost of compost, irrigation water, seed rate of 20 kg ha-1; ^2^Rental cost of 4-wheel tractor @ 2750 ha-1; ^3^Rental charge of rice transplanter; ^4^Urea @ INR 6, DAP @24, Potash @ 16 and Zinc @20 kg^-1^; ^5^Pre- and post-emergence herbicides, insecticide, and fungicide; ^6^rental cost of thresher INR @100/hr for 20 hr. ^7^Irrigation cost based on duration of irrigation x the irrigation cost @ 100 INR h^-1^ for 6.5 hp diesel pump ^8^Wage rate of INR 198 in 2014 and 204 in 2015; ^9^Exchange rate of 1USD = INR 62;

Table S6. Labor use and cost of production of maize in MW system during 2014-2015.

| Input and operation | Labor requirement (no. person-days ha^-1^) | | Input & machinery cost (USD ha^-1^) | | Total cost of each activity  (USD ha^-1^) | |
| --- | --- | --- | --- | --- | --- | --- |
|  | 2014 | 2015 | 2014 | 2015 | 2014 | 2015 |
| Hybrid seed^1^ |  |  | 16 | 16 | 16 | 16 |
| Land preparation^2^ |  |  | 44 | 44 | 44 | 44 |
| Line seeding | 7 | 7 |  |  | 22 | 22 |
| Fertilizer (Urea, DAP, Potash)^3^ | 3 | 3 | 104 | 104 | 114 | 114 |
| Weed control^4^ | 10 | 10 | 49 | 49 | 81 | 82 |
| Harvesting^5^ | 15 | 15 |  |  | 48 | 49 |
| Shelling, cleaning and drying | 3 | 3 | 39 | 39 | 48 | 49 |
| Total No. of labor | 35 | 35 |  |  |  |  |
| *Laser levelling and bed formation* |  |  |  |  |  |  |
| Levelling at 0% |  |  | 160 | 160 | 160 | 160 |
| Levelling at 0.1% |  |  | 45 | 45 | 45 | 45 |
| Bed formation |  |  | 34 | 34 | 34 | 34 |
| *Irrigation ^6^* |  |  |  |  |  |  |
| Farmer levelling (FL) | 2 | 2 | 49 | 101 | 55 | 108 |
| 0% Flat | 2 | 2 | 41 | 97 | 47 | 104 |
| 0.1% Flat | 2 | 2 | 32 | 85 | 38 | 91 |
| 0% Bed | 2 | 2 | 30 | 65 | 36 | 72 |
| 0.1% Bed | 2 | 2 | 24 | 60 | 30 | 67 |
| *Total labor, input, and production costs* |  |  |  |  |  |  |
| Farmer levelling (FL) | 112 | 115 | 301 | 354 | 429 | 484 |
| 0% Flat | 112 | 115 | 453 | 509 | 581 | 640 |
| 0.1% Flat | 112 | 115 | 489 | 542 | 617 | 673 |
| 0% Bed^7^ | 112 | 115 | 316 | 351 | 604 | 642 |
| 0.1% Bed^7^ | 112 | 115 | 515 | 551 | 643 | 682 |

^1^25 kg ha^-1^ seed rate @40 kg^-1^; ^2^Rental charge of 4-wheel tractor ha^-1^; ^3^Urea @6, DAP @24, Potash @ 17 kg^-1^; ^4^Pre- and post-emergence herbicides; ^5^Rental cost of thresher cost @800 h^-1^; ^7^Irrigation cost based on duration of irrigation x the irrigation cost @ 100 INR h^-1^ for 6.5 hp diesel pump; ^7^includes the cost of laser levelling plus bed formation.

Table S7. Labor use and cost of production of wheat in both RW and MW during 2014-2016.

| Input and operation | Labor requirement (no. person-days ha^-1^) | | Input & machinery cost (USD ha^-1^) | | Total cost of each activity | |
| --- | --- | --- | --- | --- | --- | --- |
|  | 2014 | 2015 | 2014 | 2015 | 2014 | 2015 |
| Seed^1^ |  |  | 79 | 79 | 79 | 79 |
| Seeding^2^ | 1 | 1 | 32 | 32 | 35 | 35 |
| Fertilizer (Urea, DAP, Potash)^3^ | 3 | 3 | 104 | 104 | 114 | 114 |
| Weed control^4^ | 2 | 2 | 49 | 49 | 55 | 55 |
| Harvesting | 25 | 25 |  |  | 80 | 82 |
| Threshing^5^ |  |  | 39 | 39 | 39 | 39 |
| Winnowing | 3 | 3 |  |  | 10 | 10 |
| *Irrigation cost in rice-wheat system^6^* |  |  |  |  |  |  |
| Farmer levelling (FL) | 4 | 4 | 248 | 184 | 260 | 197 |
| 0% Flat | 4 | 4 | 186 | 159 | 199 | 172 |
| 0.1% Flat | 4 | 4 | 174 | 139 | 186 | 153 |
| *Irrigation cost in maize-wheat system* |  |  |  |  |  |  |
| Farmer levelling (FL) | 4 | 4 | 213 | 117 | 226 | 130 |
| 0% Flat | 4 | 4 | 192 | 107 | 205 | 120 |
| 0.1% Flat | 4 | 4 | 190 | 101 | 203 | 114 |
| 0% Bed | 4 | 4 | 186 | 88 | 198 | 101 |
| 0.1% Bed | 4 | 4 | 178 | 82 | 191 | 96 |
| Total No. of labor | 38 | 38 | - |  |  |  |
| *Total labor, input and production cost of wheat in RW system* |  |  |  |  |  |  |
| Farmer levelling (FL) | 121 | 125 | 551 | 487 | 672 | 612 |
| 0% Flat | 121 | 125 | 489 | 462 | 610 | 587 |
| 0.1% Flat | 121 | 125 | 477 | 443 | 598 | 568 |
| *Total labor, input and production cost of wheat in MW system* |  |  |  |  |  |  |
| Farmer levelling (FL) | 121 | 125 | 516 | 420 | 638 | 545 |
| 0% Flat | 121 | 125 | 495 | 410 | 617 | 535 |
| 0.1% Flat | 121 | 125 | 493 | 404 | 614 | 529 |
| 0% Bed | 121 | 125 | 489 | 391 | 610 | 516 |
| 0.1% Bed | 121 | 125 | 481 | 386 | 602 | 511 |

^1^120 kg ha^-1^ seed rate, seed treatment @ 100 ha^-1^; ^2^Rental charge of 4-wheel tractor drawn zero tillage seed-cum-ferti-drill @2000 ha^-1^; ^3^Urea @6, DAP @24, and Potash @ 17 kg^-1^; ; ^4^Pre and post-emergence herbicides; ^5^Rental cost of thresher cost @800 h^-1^; ^6^Irrigation based on actual time recorded in each irrigation x the irrigation cost @ 100 INR h^-1^ for 6.5 hp diesel pump.

Figure S1. Soil water tension at 15 cm soil depth in rice (a) and at 32.5 cm depth in wheat in RW system (b), maize (c) and wheat in MW system (d). Data are from 0%Flat treatment from one replicate for all crops in 2014-15. Data are the average of readings at 3 locations in each plot. standard error over the growing season was 13% of the mean in rice and 18% of the mean in wheat in rice-wheat system and 14%% of the mean in maize and 17% of the mean in wheat in maize-wheat system.

Figure S2. Grain yield of each crop in the head, middle and tail sections of each treatment of the rice-wheat (RW) and maize-wheat (MW) systems. The graded colour bar to the right of each figure is the legend for yield in kg ha^-1^.

Figure S3. Volumetric moisture content (VMC; %) before and after irrigation in different treatments in rice-wheat system.

Figure S4. Volumetric moisture content (VMC; %) before and after irrigation in different treatments in maize-wheat system.
